# Pictures of social interaction prompt a sustained increase of the smile expression and induce sociability

**DOI:** 10.1038/s41598-021-84880-9

**Published:** 2021-03-09

**Authors:** Bruna Eugênia Ferreira Mota, Paula Ohana Rodrigues, Kíssyla Christine Duarte Lacerda, Isabel Antunes David, Eliane Volchan, Rafaela Ramos Campagnoli, Gabriela Guerra Leal Souza

**Affiliations:** 1 Laboratory of Psychophysiology, Department of Biological Sciences, Federal University of Ouro Preto, Ouro Preto, 35400000 Brazil; 2 School of Nutrition, Federal University of Ouro Preto, Ouro Preto, 35400000 Brazil; 3 Department of Neurobiology, Institute of Biology, Fluminense Federal University, Niterói, 24020141 Brazil; 4 Department of Physiology and Pharmacology, Biomedical Institute, Fluminense Federal University, Niterói, 24210130 Brazil; 5 Institute of Biophysics Carlos Chagas Filho, Federal University of Rio de Janeiro, Rio de Janeiro, 21941902 Brazil

**Keywords:** Emotion, Peripheral nervous system, Social behaviour, Social neuroscience

## Abstract

Viewing pictures of social interaction can facilitate approach behaviors. We conducted two studies to investigate if social interaction cues, empathy, and/or social touch modulate facial electromyographic (EMG) reactivity (as evidenced by the zygomaticus major and corrugator supercilii muscles) and mood states. We presented bonding pictures (depicting social interaction) and control pictures (without social interaction) while continuously recording zygomatic and corrugator EMG activities. In both studies, picture blocks were paired by valence and arousal. All participants were college students. In study 1, participants (n = 80, 47 women) read relevant priming texts immediately before viewing each block of 14 pictures. In study 2, participants did not read (n = 82, 63 women) priming texts before each block of 28 pictures. In study 1 and study 2, participants also completed mood states questionnaires to assess sociability and altruistic behavior. Empathy and social touch frequency were also assessed by self-reported questionnaires. In both studies, bonding pictures increased the zygomatic activity and the self-reported sociability feeling compared to control pictures. Only in study 2, bonding pictures decreased median corrugator activity compared to control pictures. We concluded that social interaction cues were efficient to increase sociability and prompt a sustained smile expression regardless of priming texts.

## Introduction

Social contexts can lead to positive emotions, and engaging in social interaction is strongly associated with smiling^1^. According to the Simulation of Smiles Model^2^, one essential social function of smiling is to indicate positive social motives, which can be seen as the affiliative smile. This facial expression, similar to social touch-like behaviors, can enhance the ability to create groups and social bonds, overcoming the limitation of expanding social bonds that depend on physical contact^3,4^. Further, Golland et al.^5^ demonstrated that the smiling expression, assessed by zygomaticus major electromyographic (EMG) activity, can be a social facilitator and is contagious in a social environment, further predicting positive affiliative feelings, like empathy. Although Hess and Fischer^6^ argued that the emotional response to emotionally charged faces occurs only if there is an affiliative intent between observers and those expressing the emotion, others have proposed that even with a remote possibility of social acceptance gain, smiling can occur as an automatic behavior^1,7^. Thus, smiling occurs automatically and may have affiliative features.

Visual affiliated stimuli, such as pictures of families, serve as powerful social stimuli and are rated as highly pleasant and evoked the highest EMG activity in the zygomaticus major muscle compared to other picture categories (e.g., adventure, erotic, nature, objects, pollution, incidents)^8^. Social bonding stimuli, such as dyads interacting, are perceived as more pleasant and arousing than non-bonding stimuli, such as dyads not interacting^9^. During positive social conditions, such as achieving a higher social status or viewing dyads happily interacting, individuals tend to present higher zygomatic EMG activity but lower corrugator EMG activity (related to frowning)^10,11^. In addition, during negative social conditions, as getting lower social status or viewing dyads in an angry interaction, the corrugator activity is enhanced but the zygomatic activity decreases as a counterpart^10,11^.

Previous research have failed to disentangle the different features of pleasant social stimuli. Furthermore, how social stimuli affect facial expressions and mood states is still a matter of debate. This study investigated the influence of social stimuli on preparatory psychophysiological response and affiliation motivations using tightly controlled stimuli balanced on emotional ratings (valence and arousal) and smiling. To the best of our knowledge, this is the first study that compares the reactivity from facial muscles, i.e., zygomaticus major and corrugator supercilii, evoked by well-controlled pleasant social stimuli, where pictures comprising the same dyads solely differ by the social bonding context (i.e., with or without social interaction). Therefore, we aimed to investigate whether social interaction pictures modulate facial electromyographic reactivity and mood states through two studies. In study 1, we investigated whether zygomatic and/or corrugator EMG activity, as well as mood states, could be modulated by well-controlled bonding and non-boding (control) pictures previously contextualized by priming texts, and if the EMG activity could be associated with self-reported empathy and social touch levels. As a further goal, study 2 was conducted to expand and strengthen study 1 findings by presenting the same bonding and control stimuli in the absence of priming texts, removing a potential confounding factor between the contextual prime and stimulus category. Associations between self-reported empathy and social touch levels and facial EMG activities are also sought.

We expect that inferring an interaction between other people would be extremely relevant to human beings (and other social species). Further, such feature may be sufficient to elicit a preparatory and/or sustained affiliative response reflected in the smile expression and self-reported emotion measures. Therefore, we hypothesized a higher reactivity of the zygomaticus major muscle and higher sociability and altruistic behavior upon exposure to bonding pictures when compared to exposure to control pictures. Less fear of rejection is also expected. The corrugator supercilii muscle, in turn, would not exhibit any significant EMG reactivity or would present lower reactivity upon exposure to bonding stimuli compared to control stimuli. Moreover, empathy and social touch (mutual grooming) could modulate facial EMG reactivity when viewing bonding scenes. We hypothesized that all emotional responses to viewing social interaction stimuli (bonding pictures), compared to non-social interaction stimuli (control pictures), would be strengthened by priming texts of social interaction or isolation-related content.

## Methods

### Study 1

#### Participants

Eighty undergraduate and graduate students (47 women and 33 men) from the Federal University of Ouro Preto, aged 18–35 years old (mean = 23.2, SD = 3.0), participated in this study. Participants were non-smokers, reported no mental disorders, and were not taking medication (except contraceptives). All participants provided written informed consent. The study was conducted in accordance with the Declaration of Helsinki. The protocol was approved by the Research Ethics Committee of the Federal University of Ouro Preto (CAAE: 32885314.2.0000.5150). Data were collected before the COVID-19 outbreak.

#### Visual stimuli

Participants were exposed to three blocks of visual stimuli—training, bonding, and control blocks—comprising a priming text (see Supplementary Material: Priming Texts) followed by pictures. The training block included four pictures of objects (7002, 7004, 7009, 7010) from the International Affective Pictures System’s (IAPS) catalogue^12^, which were intended to familiarize participants with the experiment dynamics and were not included in the analysis.

**Figure 1:**
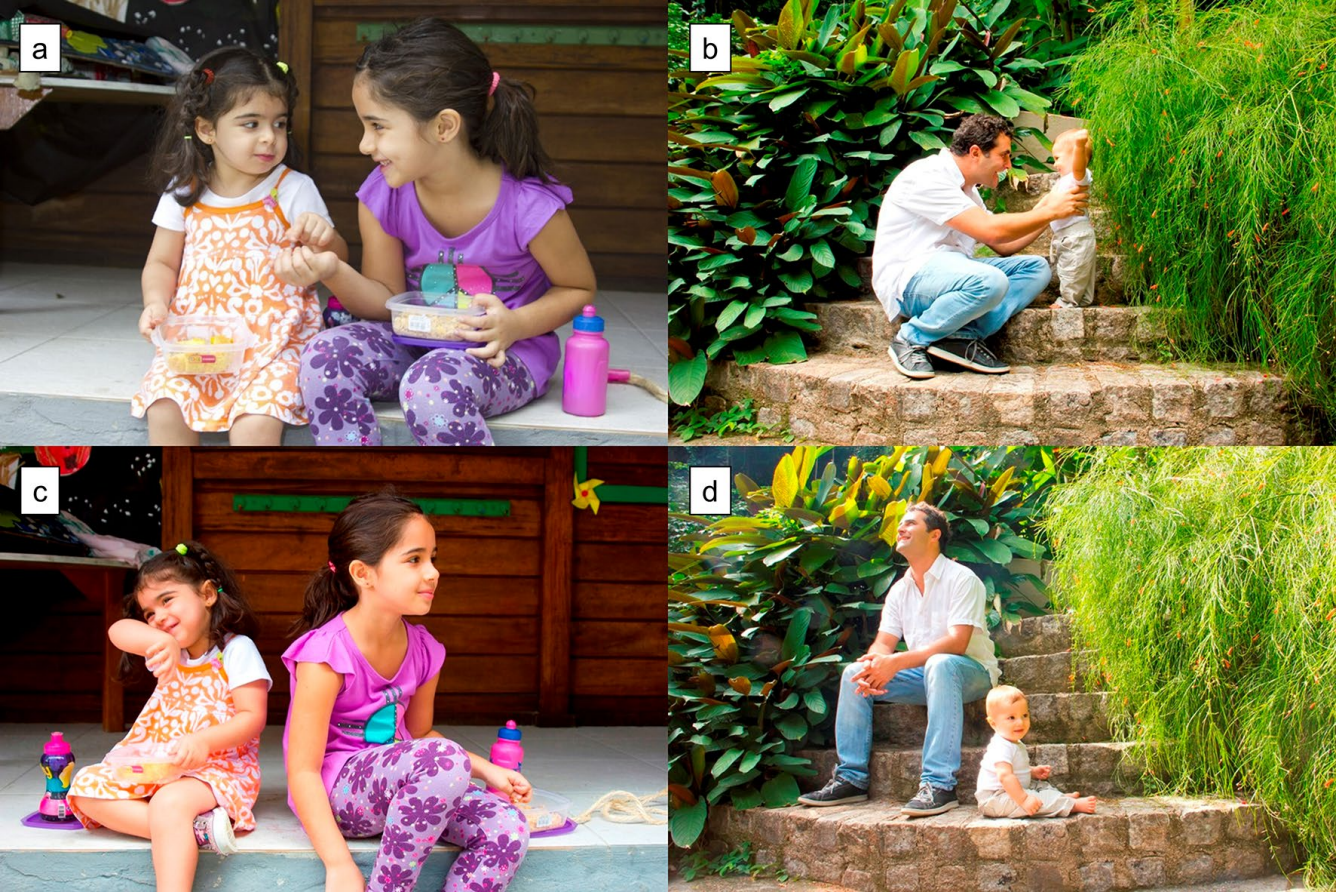
Examples of bonding and control pictures. *Note* Examples of bonding pictures (**a** and **b**) and control pictures (**c** and **d**). For more details about the complete picture catalogue and its standardization, see Silva et al.^3^. All pictures from the catalogue are property of the present research group, and their reproduction is authorized for scientific purposes only.

Bonding and control pictures were selected from a catalogue of pictures our research team previously created to study visual stimuli with and without social interaction content. All pictures were classified by valence and arousal^9^, following IAPS’s^12^ normative instructions (see Silva et al.^9^ for more details about the catalogue and classification; for examples of stimuli used in this project, see Fig. 1). Each picture block, either bonding or control, consisted of 14 pictures of the same category. All pictures displayed two people (child + child or child + adult) and were grouped in pairs. Each picture pair presented the same dyad against the same background, with the dyad interacting (bonding) in one and not interacting (control) in the other category. Bonding and control blocks presented the same number of children and a similar amount of pictures of people smiling (bonding 50%, control 43%). Pictures were presented randomly within each block, and presentation order was counterbalanced across participants. Mean valence (*M*_Bonding_ = 7.15, SD = 0.44; *M*_Control_ = 7.03, SD = 0.42; *t* = 0.68, p = 0.50) and arousal (*M*_Bonding_ = 3.75, SD = 0.46; *M*_Control_ = 3.92, SD = 0.67; *t* =  − 0.81, p = 0.42) for the bonding and control blocks were equivalent.

Each block began with a priming text (neutral, social interaction-related, or social isolation-related) to allow participants to contextualize the upcoming pictures, considering the presence or absence of social interaction in the pictures (see Supplementary Material: Priming Texts).

#### Empathy and mutual grooming traits

To assess empathy and the frequency of social touch (allo-grooming), participants completed the following questionnaires:

*Emotional Contagion Scale* (ECS)^13^: 18 items evaluate people’s tendency to consider or be influenced by others’ emotions. Higher scores indicate greater emotional contagion.

*Interpersonal Reactivity Index* (IRI)^14^: 26 items evaluate different dimensions of empathy, including cognitive and affective components. Higher scores indicate more empathic people.

*Social Touch Scale or Mutual Grooming*^15^: 28 items, of which half (14) relate to the frequency with which a person receives social touch, while the other half addresses the frequency with which a person performs social touch. Higher scores indicate a higher frequency of social touch in general.

#### Mood states scales: affiliative states and altruistic behavior

To assess mood (emotional) states, participants completed the following questionnaires:

*Affiliative State Scale*^16^: 27 adjectives form a self-reported scale that evaluates individuals’ motivational state, including a subscale that evaluates hope for closeness, which reflects the motivation to approach people, and another for fear of rejection, which reflects the motivation to avoid others.

*Altruistic Behavior Scale*^17^: 16 items relate to altruistic behaviors; 8 towards a friend and 8 towards a stranger. Individuals’ subscales scores are summed to determine the total altruistic score.

#### Exhibition of visual stimuli and signal processing

All visual stimuli were displayed in full screen on a 23-inch TV monitor placed 94 cm in front of the participants. E-Prime 2.0 (Professional Psychology Software Tools Inc., Pittsburgh, PA) generated the visual stimuli shown on screen, as well as triggers related to the stimuli’s onset. The triggers were sent by cable to the electromyographic signal acquisition system (BIOPAC MP100, Biopac Systems, Inc., Goleta, CA).

BIOPAC MP100 amplifier was used to acquire EMG signals with a sampling rate of 1000 Hz with a gain of 1000 for the two connected EMG100C electromyographic modules. EMG signals were filtered online, using a 10-Hz high-pass filter and a 500-Hz low-pass filter. The amplifier was connected directly to Acknowledge software in order to acquire the real-time raw electromyographic signals. MATLAB 7.0 software (MATricesLABoratory) was used to preprocess the raw signal. The electromyographic signals of each participant were rectified, and data sheets were created using Microsoft Excel 2016.

The analysis windows of zygomaticus major and corrugator supercilii muscles were defined from − 2 to 8 s, with time zero representing the beginning of visual stimulation. Mean activation of EMG muscles activities in the interval between − 2 s and time zero (beginning of the stimulus) was used as the baseline and was applied for baseline correction. For data processing, the average amplitude of the signal was calculated in bins of 500 ms after the beginning of the stimulus, and lasted 8 s; the first 4 s represent picture viewing itself, and the subsequent 4 s represent a blank screen (i.e., the intertrial period) following Vico et al.^18^ and Guerra et al.^19,20^ procedures. For each event, the average amplitude was calculated every half second compared to the baseline immediately prior to the picture^8,19^. Once the EMG data was not normally distributed, as verified by the Shapiro–Wilk test, median amplitude was then calculated across the time-bins (see Table S1 for the Shapiro–Wilk tests results, and kurtosis and skewness data).

#### Procedures

All procedures were performed in a temperature-, illumination-, and acoustic-controlled room. Participants completed two empathy scales (ECS and IRI) and were then asked to wash their faces with water and neutral soap. Next, the researcher cleaned participants’ skin with alcohol 70% and lightly exfoliated it with a paper towel for electrode placement. Silver chloride (Ag–AgCl) 4-mm electrodes were used to record the EMG activity of the corrugator supercilii and zygomaticus major muscles. Electrodes were placed at the left side of the face, two for each muscle. The ground electrode was placed in the left lateral malleolus. The positioning of the electrodes followed Fridlund and Cacioppo’s^21^ method.

**Figure 2:**
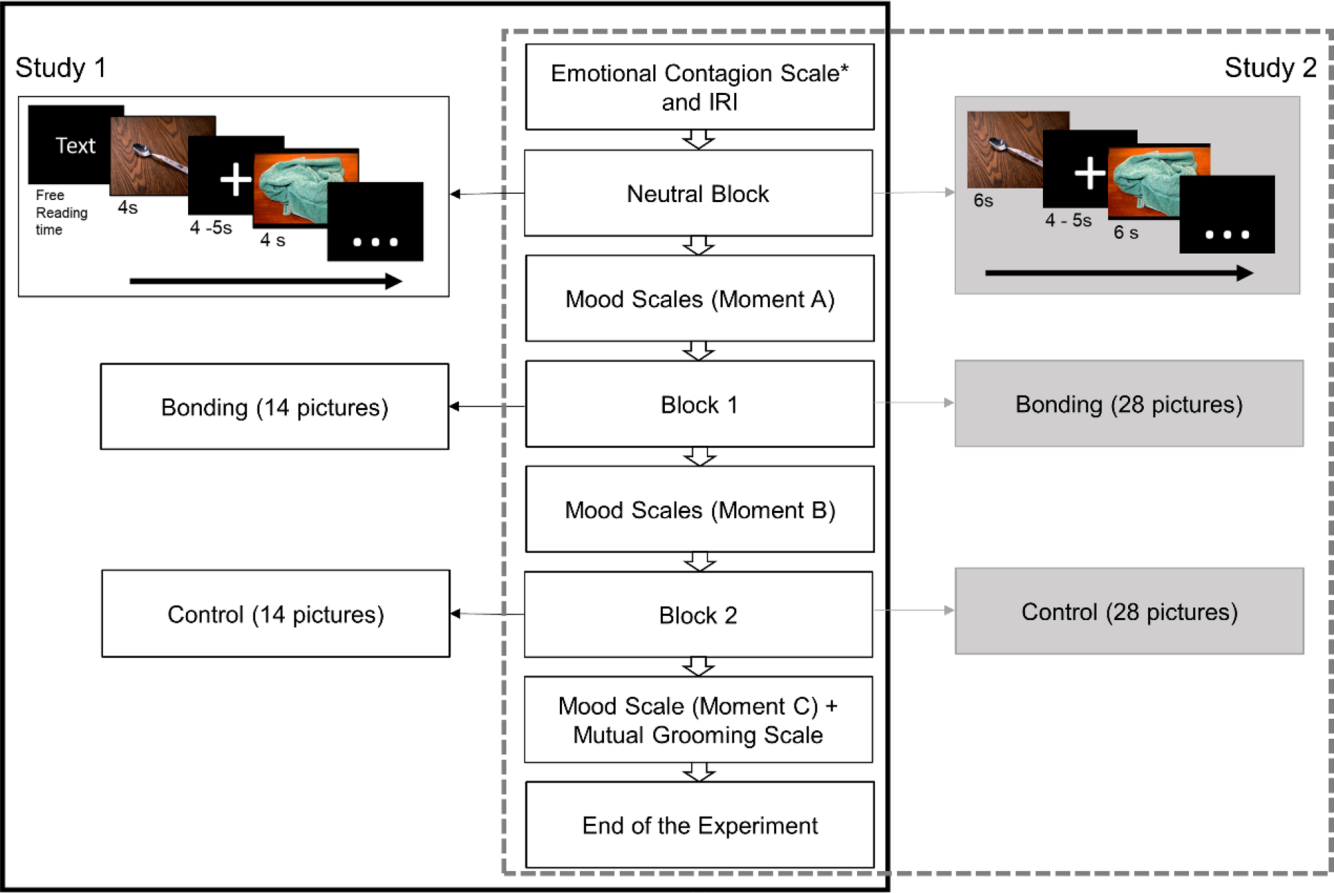
Sequence describing the order of the events throughout the experiment. *Note* This flowchart presents the sequence of the procedures for study 1 (left, black border) and study 2 (right, gray dotted border). Participants completed the empathy scales followed by viewing neutral pictures (training block). Then, mood scales were filled out (affiliative state and altruistic behavior, moment **A**). In study 1, half of the participants read a priming text (i) before viewing bonding block and control block subsequently (14 pictures each), while the other half read a priming text (ii) before viewing the control block and bonding block subsequently (14 pictures each). In study 2, participants viewed 28 bonding pictures (block 1) and 28 control pictures (block 2) without reading a priming text. In both studies, participants completed the mood scales (moment **B**) between blocks 1 and 2, as well as immediately after block 2 (moment **C**). Finally, participants completed the Mutual Grooming Scale and the experiment was concluded. All pictures were exhibited for 4 s in study 1 and for 6 s in study 2, and pictures were followed by a black screen with a fixation cross ranging from 4 to 5 s in both studies. *Emotional Contagion Scale was used only in study 1.

Participants were informed that all instructions regarding the experiment would appear on the screen. They were asked to read the texts and observe the pictures attentively. The priming texts that contextualized the pictures were shown with free reading time. For the training block, a priming text was presented followed by four IAPS neutral pictures, each remaining on the screen for 4 s and were separated by a black screen with a central fixation cross lasting randomly between 4 and 5 s. This block was not used for the EMG analysis. After this moment, participants filled out the mood state scales (Fig. 2, moment A: Altruistic Behavior and Affiliative State).

The experiment itself consisted of two blocks of emotional pictures (bonding and control), each preceded by a priming text to contextualize the pictures, with free reading time. The blocks’ presentation order (bonding/control or control/bonding) was balanced among the participants. Fourteen pictures (bonding or control) were displayed for 4 s each, separated by a black screen with a fixation cross that lasted randomly for 4 to 5 s. Figure 2 displays the sequence of the events throughout the experiment. Each block duration varied between 112 s (1 min and 52 s) and 140 s (2 min and 20 s).

After the first block, participants completed the mood state scales (Fig. 2, moment B: Altruistic Behavior and Affiliative State). Then, the experiment resumed with the second block of text and pictures, following the experimental sequence above. Participants then completed the mood state scales (Fig. 2, moment C: Altruistic Behavior and Affiliative State) and Mutual Grooming Scale. Finally, the electrodes were removed, and the participants were thanked and debriefed.

#### Statistical analyses

Statistical analyses were performed using *Statistica* 10.0 software (StatSoft, Inc.), and graphs were plotted using GraphPad Prism 8.0.1 (GraphPad Software, Inc.). Shapiro–Wilk test was run to check for data normality. See Supplementary Material (Table S1) for Shapiro–Wilk results as well as data’s skewness and kurtosis. As central tendency and dispersion measures, it is presented means (M) and standard errors (SEM) for the normally distributed data, and medians and interquartile ranges (IQR) for the non-normally distributed data.

For facial electromyography analyses, 75 participants were used (46 women and 29 men, mean age = 23.2 years, SD = 2.9) for the zygomaticus major muscle and 80 participants (47 women and 33 men; mean age = 23.2 years, SD = 3.0) for the corrugator supercilii muscle. Five participants were excluded from zygomatic EMG analysis because they were considered outliers (mean ± 3 × standard deviation). Since electromyographic data were non-normally distributed, medians of facial EMG activity during the window analysis and picture presentation were calculated for each participant. Friedman repeated-measures Analyses of Variance (ANOVAs) were used to compare muscle reactivity for bonding and control conditions during the 8-s analysis window (4 s of picture viewing followed by 4 s of blank screen; divided into 16 bins of 0.5 s each); follow-up analysis employed the Wilcoxon test for paired samples to compare the 16 bins within each sequence and median facial EMG activity during the window analysis.

Spearman’s correlation coefficients were used to investigate the associations between the median EMG activities during the 8-s window analysis and empathy (ECS and IRI), and Mutual Grooming scores. Bonferroni correction for multiple comparisons was run to establish the significance level to be adopted for the correlations between the individual trait scales and the EMG reactivity, resulting in α = 0.017 (α = 0.05/3).

Furthermore, repeated-measures ANOVA and Fisher post-test were used for the Mood State Scale that assumed a normal distribution (hope for closeness), and the Friedman repeated-measures ANOVA, followed by Wilcoxon test for paired samples in the case of Mood State Scales that were non-normally distributed (fear of rejection and altruistic behavior), to compare the emotional states before (baseline: moment A) and after each picture block (moment B and C). Values correspond to the sum of scores obtained in all scales.

The level of significance adopted in this study was α = 0.05, except for the correlation coefficients when Bonferroni’s correction was applied accordingly (α = 0.017).

### Study 2

This study was extremely similar to study 1, with a few modifications which are described below.

#### Participants

Eighty-two undergraduate and graduate students (63 women and 19 men) from the Federal University of Ouro Preto, aged 18–35 years old (mean = 23.9, SD = 3.7) participated in this study following the study 1 inclusion criteria. All participants provided written informed consent. The study was conducted in accordance with the Declaration of Helsinki. The protocol was approved by the Research Ethics Committee of the Federal University of Ouro Preto (CAAE: 90012318.1.0000.5150). Data were collected before the COVID-19 outbreak.

#### Visual stimuli

Participants were exposed to three blocks with visual stimuli. Twenty-eight pictures of objects (2880, 5510, 5520, 6150, 7000, 7002, 7004, 7006, 7009, 7010, 7025, 7050, 7080, 7130, 7090, 7150, 7170, 7175, 7207, 7211, 7217, 7233, 7235, 7490, 7550, 7595, 7705, 7950) taken from IAPS^12^ were used in the training block.

Twenty-eight pictures taken from the same catalogue employed in study 1 were used in the bonding and control blocks. Mean valence (*M*_Bonding_ = 7.17, SD = 0.39; *M*_Control_ = 7.02, SD = 0.38; *t* = 1.39, p = 0.17) and arousal (*M*_Bonding_ = 3.69, SD = 0.50; *M*_Control_ = 3.92, SD = 0.59; *t* =  − 1.58, p = 0.41) for the bonding and control blocks were equivalent. No priming texts were used in study 2.

#### Traits and mood states scales

Participants filled out the following scales described in study 1 Methods section: IRI^14^, Mutual Grooming^15^, Affiliative State^16^, and Altruistic Behavior^17^. ECS^13^ was not employed herein.

#### Exhibition of visual stimuli and signal processing

Picture exhibition and signal processing were the same as in study 1, except for picture duration which was 6 s. Window analysis was expanded to 10 s, with the first 6 s representing picture exhibition and the last 4 s representing the intertrial interval (a black screen with a central fixation cross varying from 4 to 5 s).

#### Procedures

The procedures followed the study 1 methodology (Fig. 2), omitting the priming texts and EC scale. Picture duration and block length were increased in study 2 to six seconds per picture and 28 same-content pictures per block, respectively. By doubling the number of trials in each condition, we pursued an increase in the statistical power, the same way that we aimed to strengthen emotional states by making pictures last longer (increasing exhibition from 4 to 6 s). Each block lasted between 280 s (4 min and 40 s) and 308 s (5 min and 08 s).

#### Statistical analyses

Statistical analyses were as described in study 1, except for the final sample included in data analysis and the adjusted analysis time window due to longer picture exhibition. See Supplementary Material (Table S1) for Shapiro–Wilk results as well as data’s skewness and kurtosis. After outlier removal, 78 participants (60 women and 18 men, mean age = 24.3 years, SD = 3.9) remained for zygomatic data analysis, and 80 participants (62 women and 18 men; mean age = 24.2 years, SD = 3.6) remained for corrugator data analysis. For the Friedman test, the longer 6 s picture viewing and subsequent 4 s intertrial interval (20 bins of 0.5 s each) were considered for comparisons. Last, for Spearman’s correlations, a 10 s window analysis was used for EMG signal, and Bonferroni’s correction for multiple comparisons resulted in α = 0.025 (α = 0.05/2). The level of significance adopted in study 2 was α = 0.05, except for correlation coefficients when Bonferroni’s correction was applied accordingly (α = 0.025).

## Results

### Study 1

#### Zygomatic, but not corrugator, median EMG amplitude is modulated by text-primed social interaction pictures

**Figure 3:**
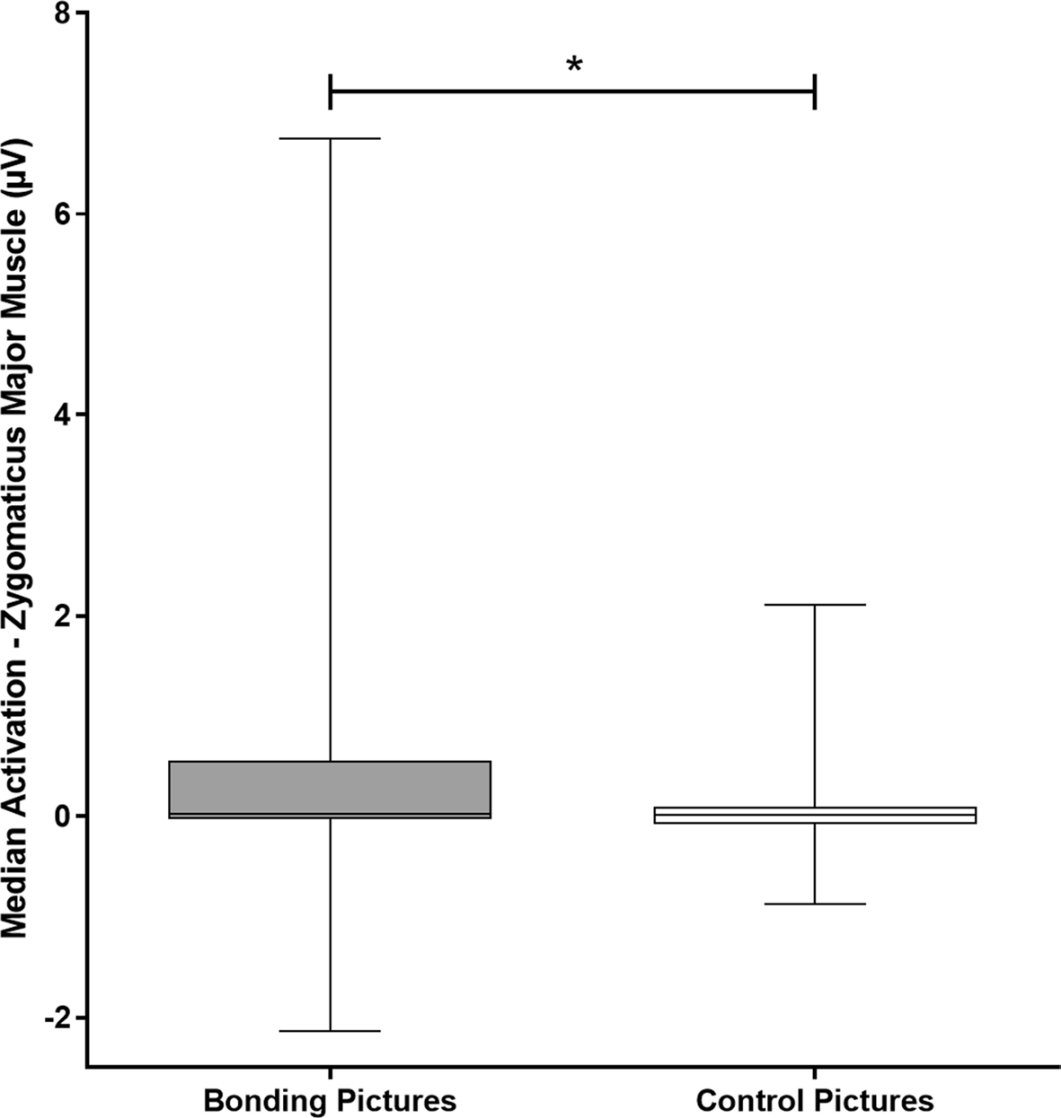
Medians and interquartile ranges of the electromyographic activity (EMG) of the zygomaticus major muscle per condition in study 1. *Note* Medians and interquartile ranges (IQR) of the electromyographic activity of the zygomaticus major muscle. The whiskers of the box represent minimum and maximum occurring data values. Median bonding = 0.29 µV, IQR =  − 0.02 to 0.53; median control = 0.01 µV, IQR =  − 0.07 to 0.10; p = 0.045; *p < 0.05.

Considering the whole analysis window, zygomatic EMG amplitude was greater during bonding than control block when both picture sets were primed by content-related text (Median_*Bonding*_ = 0.29 µV, IQR =  − 0.02 to 0.53; Median_*Control*_ = 0.01 µV, IQR =  − 0.07 to 0.10; p = 0.045) (Fig. 3). Considering only picture presentation (i.e., from 0 to 4 s), similar results were found (Median_*Bonding*_ = 0.03 µV, IQR =  − 0.02 to 0.53; Median_*Control*_ = 0.01 µV, IQR =  − 0.07 to 0.57; p = 0.045).

Then, in a follow-up analysis, we investigated the temporal course of zygomatic muscle activity along with trials, i.e., during picture viewing (4 s) and after picture offset (4 s intertrial interval), following Vico et al.^18^ and Guerra et al.^19,20^ procedures. There was a significant difference in zygomatic activity over time when comparing bonding and control blocks (χ^2^ = 108.61; p < 0.0001). There was also a difference in zygomatic activity during bonding picture exhibition over time (χ^2^ = 80.01; p < 0.0001). However, no changes were found in zygomatic activity during the control block (χ^2^ = 10.99; p = 0.75). For all time bins post-hoc comparisons, please see Supplementary Material Table S2 (within block comparisons block) and Table S3 (between blocks comparisons).

**Figure 4:**
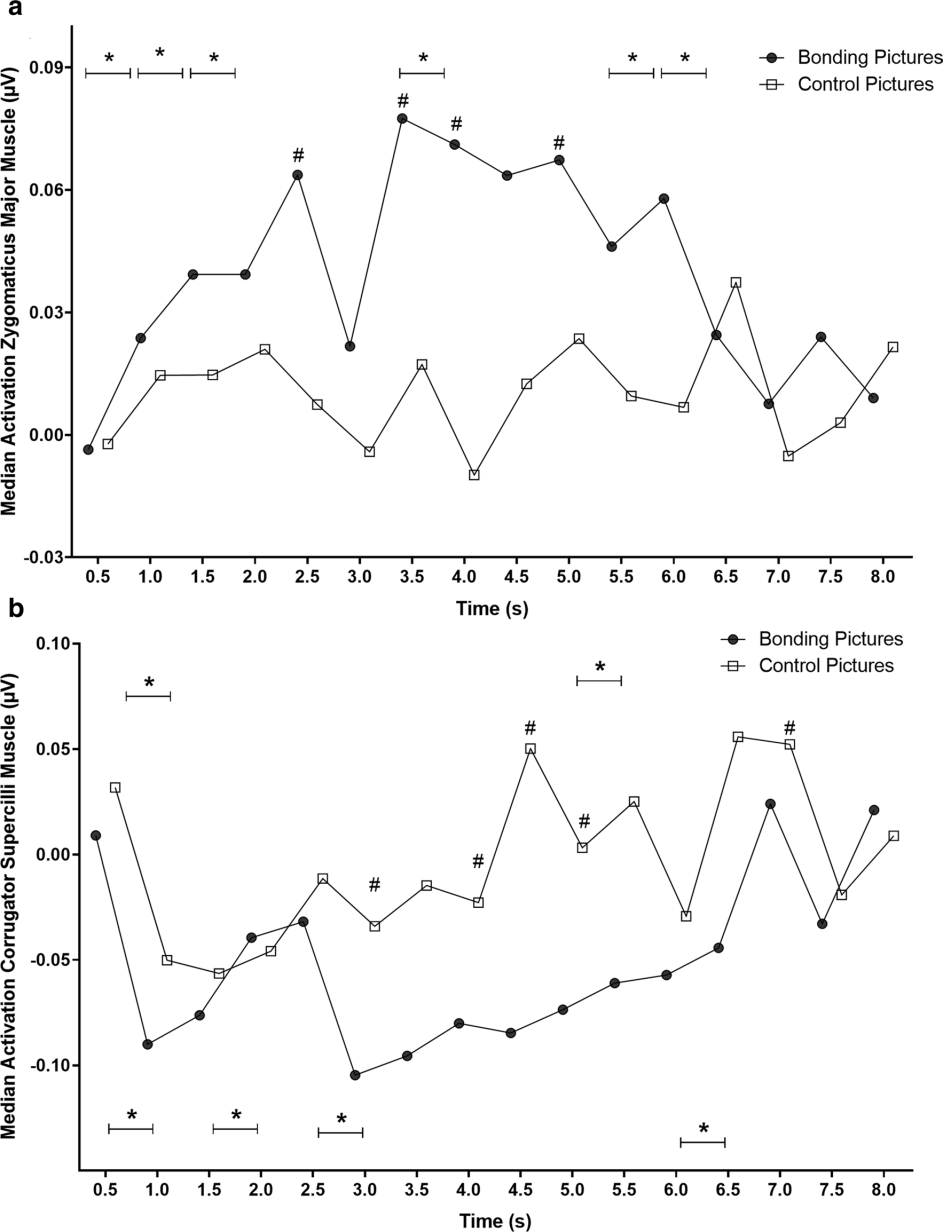
Temporal course of the electromyographic activities (EMG) of the zygomaticus major and corrugator supercilli muscles in study 1. *Note* The pictures were displayed on the screen between 0 and 4 s. From 4 s to the end, the intertrial interval occurs. Note that EMG activities are sustained throughout the trial. (**a**) Asterisks on the top of the plot represent differences in consecutive time bins for bonding pictures. (**b**) Asterisks on the top of the plot indicate differences in consecutive time bins for control pictures; asterisks at the bottom of the plot indicate differences in consecutive time bins for bonding pictures. #p < 0.05 represents significant differences between conditions. *p < 0.05 represents significant differences within condition.

Comparing bonding and control conditions at the same moment (between blocks comparisons), the EMG activity of the zygomatic muscle was higher for the bonding block than for the control block specifically on 2.5 s, 3.5 s, 4.0 s, and 5.0 s (Fig. 4a). Throughout the zygomatic analysis window, EMG activity within bonding block showed a significant difference between consecutive seconds from the beginning of picture presentation to 2.0 s and from 3.5–4.0 s. After picture offset, a significant difference was detected from 5.5 to 6.5 s (Fig. 4a). Data dispersions regarding these analyses are represented in Figure S1a in Supplementary Material.

For the corrugator supercilii muscle, there was no difference between the median values of EMG amplitude for bonding and control blocks during the whole analysis window (Median_*Bonding*_ =  − 0.056 µV, IQR =  − 0.33 to 0.06; Median_*Control*_ =  − 0.017 µV, IQR =  − 0.20 to 0.09; p = 0.11). During picture presentation (i.e., from 0 to 4 s), similar results were found (Median_*Bonding*_ =  − 0.056 µV, IQR =  − 0.33 to 0.06; Median_*Control*_ =  − 0.017 µV, IQR =  − 0.0 to 0.09; p = 0.11).

There was a significant difference in corrugator activity over time, when comparing the time bins between bonding and control blocks (χ^2^ = 122.36; p < 0.0001). There was also a difference in corrugator activity during bonding (χ^2^ = 108.71; p < 0.0001) and control (χ^2^ = 37.19; p = 0.01) picture exhibition. For all time bins post-hoc comparisons, please see Supplementary Material Table S2 (within block comparisons) and Table S3 (between blocks comparisons).

Comparing bonding and control conditions at the same moment (between blocks comparisons), corrugator EMG activity was lower for the bonding block than for the control block specifically on 3.0 s, 4.0 s, 4.5 s, 5.0 s, and 7.0 s (Fig. 4b). Throughout the corrugator analysis window, EMG activity within bonding block showed a significant difference between consecutive seconds during picture presentation specifically for 0.5–1.0 s, 1.5–2.0 s, and 2.5–3.0 s time bins. After picture offset, a difference was detected from 6.0–6.5 s (Fig. 4b). Within control block, differences over time in corrugator activity were observed between 0.5–1.0 s and 5.0–5.5 s (Fig. 4b). Data dispersions regarding these analyses are represented in Figure S1b in Supplementary Material.

#### Bonding pictures primed by a social interaction-related text increase the hope for closeness and altruistic behavior

**Figure 5:**
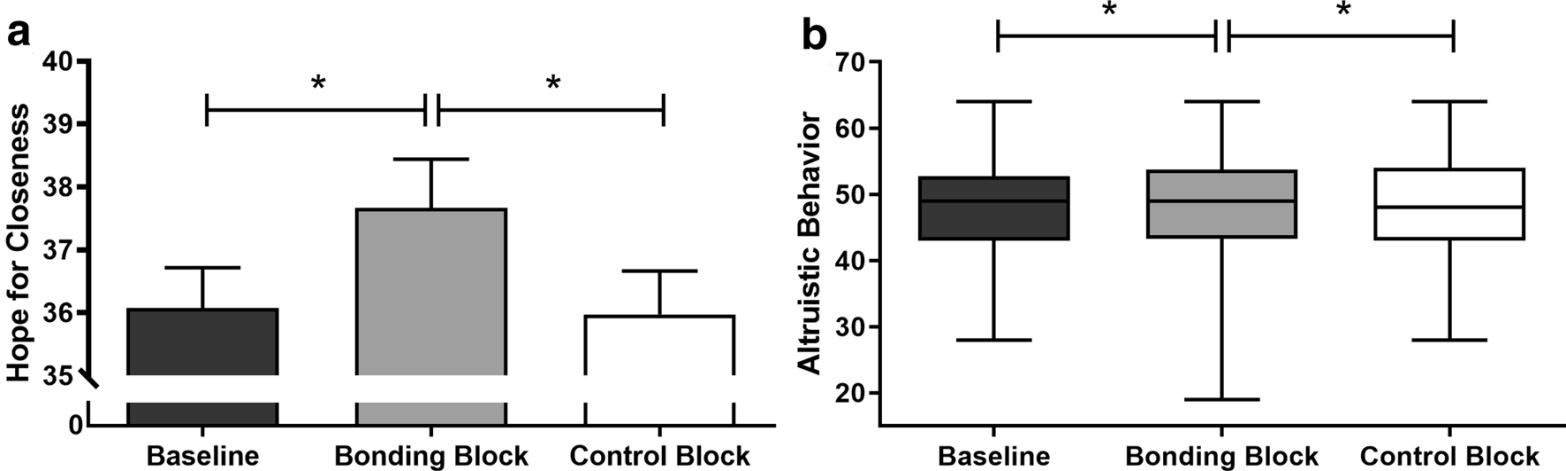
Mood state scores in study 1. *Note* (**a**) Self-reported hope for closeness assessed before (baseline) and after bonding and control blocks. Bonding pictures promoted greater hope for closeness compared to baseline and to control block levels. Error bars indicate standard error. (**b**) Altruistic behavior assessed before (baseline) and after bonding and control blocks. Bonding pictures promoted greater altruistic behavior when compared to baseline and to control block levels. Box extends from the 25th to 75th percentiles (interquartile range) and the error bars indicate minimum and maximum values. *p < 0.05.

Altruistic Behavior Scale and Affiliative State (fear of rejection and hope for closeness dimensions) were used to evaluate if participants’ mood states would be changed upon exposure to the bonding and control blocks, which were primed by a social interaction-related text. There was a significant difference in hope for closeness across different moments (F_(2, 158)_ = 6.43; p = 0.002). Post-test showed an increase in hope for closeness after exposure to the bonding block (M = 37.66, SEM = 0.78) compared to baseline (M = 36.07, SEM = 0.64, p = 0.003) and control block levels (M = 35.97, SEM = 0.69; p = 0.001). No difference was found between baseline and the moment after exposure to the control block (p = 0.85) (Fig. 5a). For fear of rejection, there was no significant differences across the different moments (χ^2^ = 1.521; p = 0.467).

Regarding Altruistic Behavior Scale, we found differences across different moments (χ^2^ = 11.41, p = 0.003). Participants reported a significant increase in altruistic behavior after exposure to the bonding block (Median_*Bonding*_ = 49.0, IQR = 44.0–54.0) compared to baseline (Median_*Baseline*_ = 49.0, IQR = 43.0–52.0, p = 0.003) and control block (Median_*Control*_ = 48.00, IQR = 43.00–54.00, p = 0.02) (Fig. 5b). Surprisingly, even though the absolute median values of altruistic behavior are the same at baseline and after the bonding block, the Wilcoxon matched-pairs test—used for this comparison—demonstrated a significant difference. Therefore, individuals self-reported higher altruism after observing bonding pictures when compared either to control pictures or to baseline levels.

#### Empathy and social touch are associated to zygomatic reactivity to bonding pictures primed by a social interaction-related text

After checking the influence of the bonding and control pictures primed by content-related text in facial EMG activities, we then evaluated whether individual traits would affect the EMG reactivity. For a full data table showing all possible association comparisons see Supplementary Material (Table S4).

We found positive correlations between zygomatic amplitude during bonding picture viewing and Global Empathy (IRI) (rho = 0.30; p = 0.012) and the frequency of social touch (rho = 0.31; p = 0.008), but not to ECS (rho = 0.18; p = 0.13). There were no significant correlations between zygomatic EMG activity and the above referred self-reported scales upon exposure to the control block primed by a social isolation-related text. Thus, the more evident is the social characteristics of the individual, i.e., presenting a higher empathy trait and usually performing/ receiving more social touch, the higher the observed smiling expression during the bonding block.

For corrugator, there were no associations between individual trait scales and EMG activity during either bonding or control pictures primed by content-related texts.

### Study 2

Here we explored whether social interaction pictures would promote any modulation over zygomatic and corrugator EMG reactivity when compared to control stimuli, i.e., pictures without social interaction. Note that valence and arousal of the pictures’ sets were paired between conditions. Therefore, any differences in EMG reactivity to picture viewing would be prompted solely by social interaction features per se. Importantly, and differently from study 1, no priming texts were used, and pictures were visible for longer (6 s) and in greater number (n = 28).

#### Not only zygomatic, but also corrugator, median EMG amplitude is modulated by social interaction pictures

**Figure 6:**
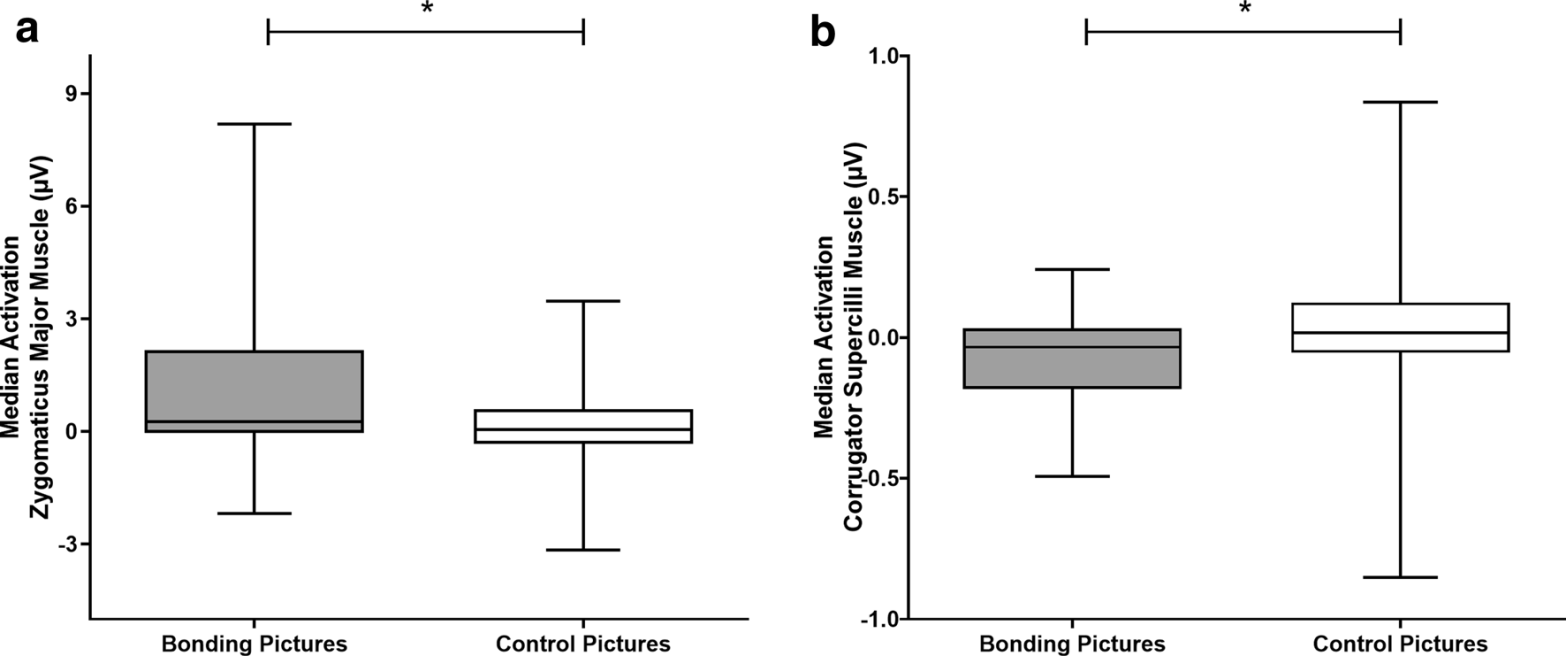
Medians and interquartile ranges of the electromyographic activity (EMG) of the zygomaticus major and corrugator supercilli muscles per condition in study 2. *Note* (**a**) Medians and interquartile ranges (IQR) of the EMG of the zygomaticus major muscle. Median bonding = 0.26 µV, IQR =  − 0.04 to 2.16; median control = 0.04 µV, IQR =  − 0.32 to 0.57; *p < 0.00001. (**b**) Medians and interquartile ranges of the EMG of the corrugator supercilli muscle. Median bonding =  − 0.034 µV, IQR =  − 0.17 to 0.03; median control = 0.016 µV, IQR =  − 0.05 to 0.12; p = 0.001. The whiskers of the box represent minimum and maximum occurring data values. *p < 0.05.

Zygomatic EMG amplitude was greater during the bonding block than the control block during the whole analysis window (Median_*Bonding*_ = 0.26 µV, IQR =  − 0.04 to 2.16; Median_*Control*_ = 0.04 µV, IQR =  − 0.32 to 0.57; p < 0.00001) (Fig. 6a). During picture presentation (i.e., from 0 to 4 s) similar results were found (Median_*Bonding*_ = 0.26 µV, IQR =  − 0.04 to 2.16; Median_*Control*_ = 0.05 µV, IQR =  − 0.32 to 0.57; p < 0.00001).

There was a significant difference in zygomatic activity over time, comparing bonding and control pictures (χ^2^ = 291.09; p < 0.00001), as well as separately during bonding (χ^2^ = 188.44; p < 0.00001) and control (χ^2^ = 73.09; p < 0.00001) picture exhibition. For all time bins post-hoc comparisons, please see Supplementary Material Table S2 (within block comparisons) and Table S3 (between blocks comparisons).

**Figure 7:**
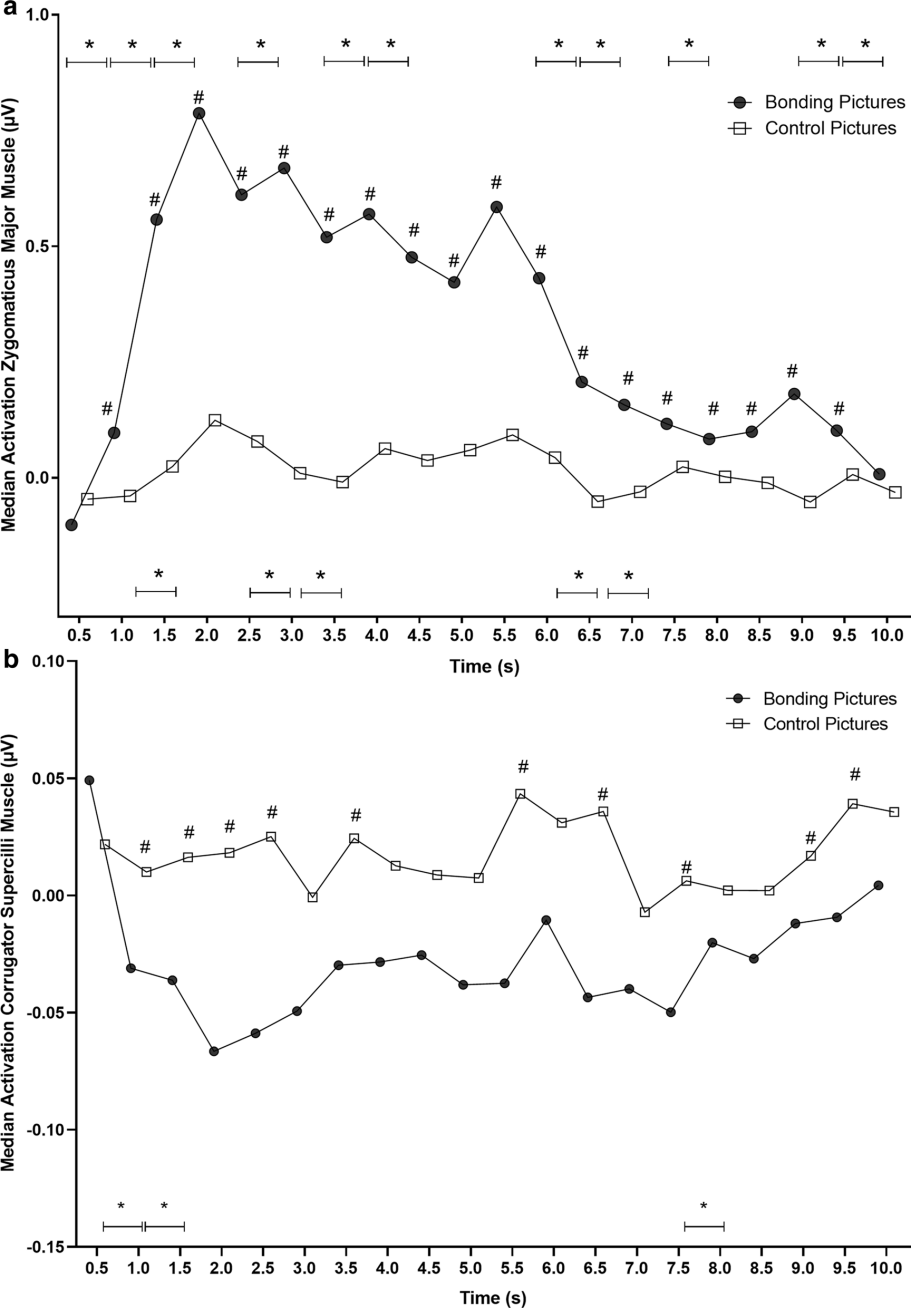
Temporal course of the electromyographic activities (EMG) of the zygomaticus major and corrugator supercilli muscles in study 2. *Note* Pictures were displayed on the screen between 0 and 6 s. From 6 s to the end, the intertrial interval occurs. Note that EMG activities are sustained throughout the trial. (**a**) Asterisks on the top of the plot represent differences in consecutive time bins for bonding pictures, while asterisks at the bottom indicate differences in consecutive time bins for control pictures. Zygomatic EMG activities between blocks comparisons were all significant except for the first and the last time bins. (**b**) Asterisks at the bottom of the plot indicate differences in consecutive time bins for bonding pictures. #p < 0.05 represents significant differences between conditions. *p < 0.05 represents significant differences within condition.

By comparing bonding and control conditions in a cross-time point fashion, a higher zygomatic EMG activity was shown for bonding pictures compared to control pictures from 1.0 s after picture onset until 9.5 s. In other words, zygomatic EMG activity was greater and sustained for bonding pictures for all the time bins, except for the very first ones and the last one (Fig. 7a).

For the follow-up analysis over time, zygomatic activity bonding pictures exhibition showed significant differences between the following time bins: 0.5–2.0 s, 2.5–3.0 s, 3.5–4.5 s, 6.0–7.0 s, 7.5–8.0 s, and 9.0–10.0 s. On the other hand, for the control pictures, fewer differences were found, specifically for these time bins: 1.0–1.5 s, 2.5–3.5 s, and 6.0–7.0 s (Fig. 7a). Data dispersions regarding these analyses are represented in Figure S2a in Supplementary Material.

For corrugator, we also found differences between the median values of EMG amplitude during bonding and control pictures analysis window (Median_*Bonding*_ =  − 0.034 µV, IQR =  − 0.17 to 0.03; Median_*Control*_ = 0.016 µV, IQR =  − 0.05 to 0.12; p = 0.001) (Fig. 6b). During picture presentation (i.e., from 0 to 4 s), similar results were found (Median_*Bonding*_ =  − 0.034 µV, IQR =  − 0.17 to 0.028; Median_*Control*_ = 0.02 µV, IQR =  − 0.05 to 0.57; p = 0.01). In addition, there was a significant difference in the corrugator activity over time, comparing bonding and control pictures (χ^2^ = 192.76; p < 0.00001) and during bonding pictures exhibition (χ^2^ = 102.70; p < 0.00001), but not during control pictures exhibition (χ^2^ = 25.17; p = 0.15). For all time bins post-hoc comparisons, please see Supplementary Material Table S2 (within block comparisons) and Table S3 (between blocks comparisons).

Corrugator EMG activity during bonding pictures exhibition showed a significant difference between the following time bins: 0.5–1.0, 1.0–1.5 s, and 7.5–8.0 s (Fig. 7b). Cross-time point comparison between conditions revealed a lower corrugator EMG activity for bonding pictures than for control pictures from 1.0 s to 9.5 s. (Fig. 7b). Thus, a sustained relaxation of the corrugator supercilii muscle was elicited by viewing bonding pictures. Data dispersions regarding these analyses are represented in Figure S2b in Supplementary Material.

#### Bonding pictures increase hope for closeness and altruistic behavior and decrease fear of rejection

**Figure 8:**
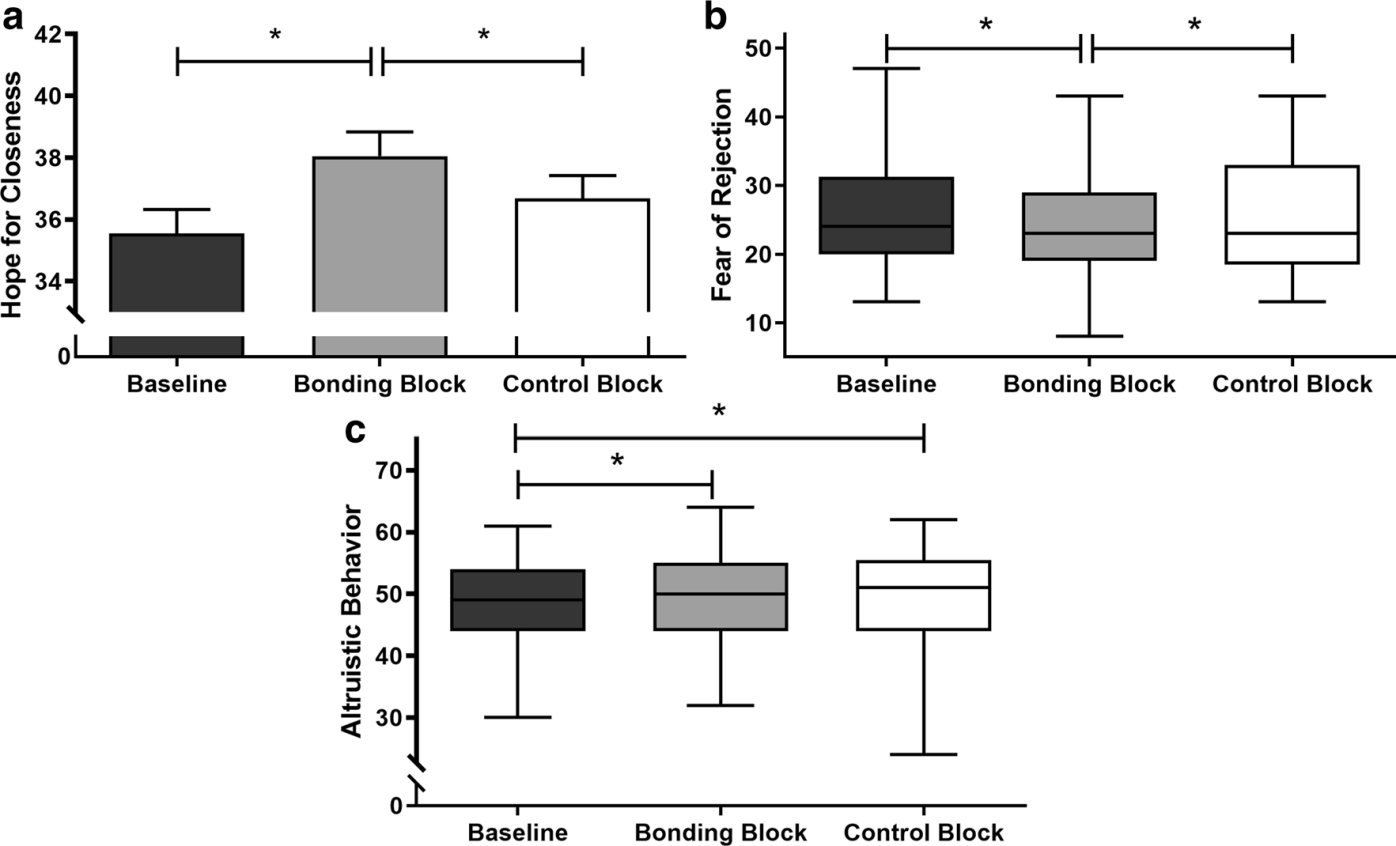
Mood state scores in study 2. *Note* (**a**) Self-reported hope for closeness assessed before (baseline) and after bonding and control blocks. Bonding pictures promoted greater hope for closeness when compared to baseline and to control block levels. Error bars indicate standard errors. (**b**) Self-reported fear of rejection assessed before (baseline) and after bonding and control blocks. Bonding pictures promoted lower fear of rejection when compared to baseline and to control block levels. Box extends from the 25th to 75th percentiles (interquartile range) and the error bars indicate minimum and maximum values. (**c**) Altruistic behavior assessed before (baseline) and after bonding and control blocks. Bonding and control pictures promoted greater altruistic behavior when compared to baseline. Box extends from the 25th to 75th percentiles (interquartile range) and the error bars indicate minimum and maximum values. *p < 0.05.

Results showed a significant difference in hope for closeness (F_(2, 160)_ = 9.09; p = 0.00018) and in fear of rejection (χ^2^ = 29.39; p = 0.009). Post-test showed an increase in hope for closeness after exposure to the bonding block (M_*Bonding*_ = 38.04, SEM = 0.78) compared to baseline (M_*Baseline*_ = 35.55, SEM = 0.77, p < 0.0001) and control block levels (M_*Control*_ = 36.68 SEM = 0.74; p = 0.013). No differences were found between the baseline and the moment after exposure to the control block (p = 0.08) (Fig. 8a). As a counterpart, we observed a decrease in fear of rejection after bonding block (Median_*Bonding*_ = 23.00, IQR = 19.00–29.00) compared to baseline levels (Median_*Baseline*_ = 24.00, IQR = 20.00–31.00; p = 0.005). In addition, fear of rejection upon exposure to bonding and to control blocks also differed (Median_*Control*_ = 23.00, IQR = 19.00–33.00, p = 0.01) (Fig. 8b).

Regarding Altruistic Behavior Scale, we found differences across the different moments (χ^2^ = 11.96, p = 0.002). Participants reported an increase in altruistic behavior after exposure to the bonding block (Median_*Bonding*_ = 50.0, IQR = 44.0–55.0) compared to baseline (Median_*Baseline*_ = 49.0, IQR = 44.0–54.0, p = 0.005). Altruism after control pictures (Median_*Control*_ = 51.00, IQR = 44.00–55.00) also increased compared to baseline (p = 0.004). No difference was found comparing the moment after exposure to bonding pictures and after control pictures (p = 0.42) (Fig. 8c).

#### Empathy, but not social touch, remains associated to zygomatic EMG reactivity to bonding pictures

During exposure to the bonding block, a positive correlation was shown between zygomatic EMG activity and the IRI empathy scale (rho = 0.28, p = 0.01). However, no association was found for the zygomatic EMG activity and the frequency of social touch (rho = 0.15; p = 0.17, assessed by the Mutual Grooming Scale). No correlations were shown between corrugator EMG activity and empathy or the frequency of social touch (IRI: rho = 0.04; p = 0.76; Mutual Grooming: rho = 0.08; p = 0.50). Additionally, no associations were found regarding control pictures and individual traits. For a full list of all correlation analyses see Supplementary Material (Table S4).

## Discussion

The present investigation used well-controlled visual stimuli with and without social interaction content (bonding and control, respectively), paired by valence and arousal to determine whether facial electromyographic reactivity and mood states would be modulated by such social content. We further investigated if empathy and frequency of social touch would modulate this process. In the presence of priming texts (study 1), bonding pictures caused greater sustained zygomatic EMG activity, hope for closeness, and altruistic behavior and decreased sustained corrugator EMG activity than control pictures. In addition, empathy levels and frequency of social touch were positively associated with zygomatic EMG activity during bonding pictures. No differences in median corrugator EMG activity or fear of rejection were found. Study 2 (without priming texts and with longer exposure to visual stimuli) replicated and strengthened some of the findings, where zygomatic EMG activity and hope for closeness and remained greater during bonding pictures compared to control pictures, and zygomatic activity was also associated with empathy during bonding picture presentation. Additionally, study 2 showed lower median and sustained corrugator EMG activity and fear of rejection during bonding pictures compared to the control pictures.

Although the majority of results were replicated in both studies, such as the sustained increase of zygomatic EMG activity and sociability due to bonding pictures stimulation, some other results differed in studies 1 and 2, e.g., the median corrugator EMG activity, fear of rejection, and the association between zygomatic activity and frequency of social touch during bonding pictures. We expected that the priming texts would potentialize the effects of the psychophysiological measures in study 1. However, even though we cannot rule out this assumption, this does not seem to be the case. Souza et al.^22^ had demonstrated that affiliative primers (pictures with social content) can improve the promotion of movements related to social behaviors, such as finger flexion. Similarly, Campagnoli et al.^23^ have shown that, when a social interaction primer (picture) is used preceding an analogous task movement, such as a caress-like movement on a soft cloth, the brain circuitry is facilitated by the pre-activation of the cortical circuit, when compared to a primer without social interaction. On the other hand, Spapé, et al.^24^ found similar results to ours by observing that the use of a tactile prime stimulus did not enhance the emotional response of physiological parameters (i.e., zygomatic and corrugator EMG activity) of individuals exposed to emotionally charged pictures. Although we have used the priming texts in study 1 aiming to strengthen the social interaction effects over the facial EMG activities and self-reported emotional assessments, it is possible that the texts acted as a confounding factor. However, this issue has been overcome in study 2.

We suggest that more prolonged exposure to bonding visual stimuli in study 2 (additional number of pictures and exposure duration) may have induced a more sustained positive emotional mood, reflected by both a decrease in median corrugator EMG activity and in fear of rejection. This modulation might have been so strong that it was no longer affected by the frequency of social touch, as seen in study 1. Exposure to a longer block of pictures can induce a sustained emotional mood^22,25^ that could reflect on the corrugator relaxation and on the decreased fear of rejection. Moreover, the activation of the corrugator supercilli muscle can be more influenced by emotional induction than the zygomatic activity^26^. Thus, the fact that the median relaxation of the corrugator was observed only in study 2 could be explained by an effective positive affect induction elicited by a more sustained emotional stimulation (higher number of pictures and longer-lasting exposure). Another possible interpretation for the significant relaxation results in corrugator median EMG activity in study 2 for bonding pictures could be an increased statistical power acquired by doubling the number of trials in each condition besides enhancing 2 s for each picture presentation.

Facial expression responses to stimuli are recognized as intimately related to valence and, to a lesser degree, to emotional arousal^27^. In other words, the higher the stimulus valence, the higher the zygomatic EMG activity is, while the lower the stimulus valence, the higher the EMG activity of the corrugator supercilii^8,11,26–28^. In this research, the bonding and control picture sets had similar valence and arousal, and the sole difference between blocks was the social interaction feature. Despite that, participants had a higher zygomatic EMG activity during the social interaction condition than control condition in both studies, regardless of a priming text. Thus, the image by itself may promote positive emotional reactions relevant to social interaction. The diminished corrugator median EMG activity occurred only in study 2, where no priming text was employed, and participants were exposed for longer to stimuli. In addition, it is noteworthy that potential group differences may occur considering block presentation order (bonding-control or control-bonding). However, we expect to have removed these potential differences by counterbalancing the block presentation order across participants.

Social scenes can facilitate approach behaviors, through the activation of the appetitive motivational system, assessed through behavioral^22^ and electroencephalographic^23^ measures. The literature also indicates that positive social interaction cues, such as situations of cooperation, are efficient to increase the activity of the zygomaticus major muscle, to decrease the activity of corrugator supercilli muscle^10^, and might also be considered as more pleasant and more arousing than stimuli without social interaction^9^. As previously mentioned, smiling plays an essential role in social life, and zygomatic activation is associated with positive social contexts^1,2,5,27,28^.

Although studies regarding the psychophysiological reactions using social interaction scenes can be found, to the best of our knowledge, these studies presented stimuli with differences in valence and arousal, which might be a confounding factor. Therefore, by detecting an increase in the zygomatic EMG activity and a decrease in the corrugator EMG activity upon exposure to scenes of positive social interaction compared to scenes comprising the same dyads paired in valence and arousal but without social interaction, this study suggests that such stimuli might foster the social effect of the smiling expression, which acts as a facilitating agent of social interactions and vice-versa^29,30^. Also, the sustained EMG activity across time, especially for the zygomaticus major muscle, has been observed previously in strongly positive stimuli, i.e., loved faces pictures^18–20^. This is corroborated by study 2 findings where, even in the absence of a text comprising social interaction content, the social visual cues per se were sufficient to elicit emotional modulations over EMG activities.

Both studies 1 and 2 showed an increase in sociability (higher score in hope for closeness sub-scale) upon bonding stimuli exposure, while a decreased fear of rejection (lower score in fear of rejection sub-scale) occurred in study 2 after viewing the bonding block when compared both to baseline and control block levels. Study 2 replicates the findings of Campagnoli et al.^23^ by lowering fear of rejection and enhancing hope for closeness after social interaction picture viewing. Moreover, an increased altruistic behavior elicited by social interaction picture viewing was observed in both studies. Altruistic behavior might be understood as a voluntary and intentional act of helping other individuals without receiving any benefit^31^. Essentially, people behave in prosocial ways, including performing altruistic acts, because they feel good doing so, as prosocial behaviors elicit positive emotions in those that perform them^32^.

The self-reported frequency of social touch (mutual grooming scores) assessed upon exposure to the bonding block is associated to the zygomatic response only under the influence of priming texts (study 1). Moreover, the zygomatic EMG activity was positively correlated to empathy in both studies. Notably, individuals with higher empathy traits tend to be more responsive to any type of facial expression and to engage more easily in prosocial behaviors^10^, and had increased zygomatic reactivity in response to happy faces^33^. In addition, social touch plays an important role in the creation and maintenance of social bonds^15,34^, as well as mitigating social exclusion feelings^35^. Souza et al.^22^ and Campagnoli et al.^23^ have shown that exposure to positive social stimuli modulate motor responses (e.g., movement of finger flexion) that are similar to social touch, suggesting that social interaction contexts are capable of preparing people for approach behaviors, such as actual social interaction. Thus, due to the relevance of empathy and social touch to social interactions, both aspects can modulate the somatic response to social interaction stimuli, but empathy appears to be particularly related to the smile expression during social interaction context, regardless of priming text. In the same way that more empathetic individuals can perceive faster and more accurately emotional displays^33^, they might be as well more responsive to social cues. As social touch and smile, we suggest that empathy possibly facilitates the engagement in positive social interactions and extends bonding ability, by enabling more empathetic individuals to be more reliable to smiling in positive social scenarios. Therefore, empathy can be seen, to a certain extent, as a facilitator of affiliative smile.

Practical implications of the present investigation include the possibility of using social interaction stimuli as a complementary therapy for individuals with social disorders (for example, social phobia). Social interaction stimuli might also be used for the promotion and increase of psychological well-being, with implications for publicity and advertising, among other areas. In light of the unprecedented situation of social isolation/distancing brought by the COVID-19 pandemic, social interaction cues might play an extremely relevant role in mental health. Therefore, strengthening social bonds even in virtual environments (online meetings, video calls, etc.) and exposure to social interaction cues might be seen as a protective factor to health. Future studies should consider neuroimaging methods to shed light on the neural processing pathways of social interaction stimuli. In addition, it would be interesting to investigate how individuals with different neurological and/or psychiatric pathologies react to social interaction scenes.

This investigation also has some caveats. The low ethnic diversity of the visual stimuli used in studies 1 and 2 could be a limitation. It is well known that feeling part of a group favors facial mimicry^36^. In 2019, more than 48% of the freshmen students in our university identified themselves as brown or black^37^. Therefore, the lack of ethnic diversity in the visual stimuli might be an important limitation of the present study since participants might have not felt completely inserted into the context. In addition, the priming texts employed in study 1 might be considered a confounding factor. However, this issue was addressed in study 2 by removing the primes (texts) and directly studying the effects of the social context over facial electromyographic activities and self-reported emotional assessments. In conclusion, this investigation showed that social interaction stimuli effectively prompt a sustained smile expression and increase sociability in humans, independent of priming texts. By using visual stimuli depicting social interaction matched by valence, arousal, number of people displayed in the scenes, and the same background as the non-social stimuli, we offer a strong contribution to the literature. By using facial electromyographic measures and self-reported questionnaires, we showed that bonding pictures prompt increased and sustained smiling expression, as well as sociability feelings and altruistic behavior, and reduce the frowning expression and the fear of rejection. Further, this investigation has unveiled that the frequency of social touch, and especially empathy, might be positively related to the facial somatic responses of the smiling expression.

## Supplementary Information

Supplementary Information
