# Supplementary material for: Pictures of social interaction prompt a sustained increase of the smile expression and induce sociability
Source: Sci Rep. 2021 Mar 9;11:5518. doi: 10.1038/s41598-021-84880-9 (PMC7943771; doi:10.1038/s41598-021-84880-9)
Supplement: Supplementary file 1 — Supplementary Information [file 41598_2021_84880_MOESM1_ESM.docx]

**Pictures of Social Interaction Prompt a Sustained Increase of the Smile Expression and Induce Sociability**

Bruna Eugênia Ferreira Mota, Paula Ohana Rodrigues, Kíssyla Christine Duarte Lacerda, Isabel Antunes David, Eliane Volchan, Rafaela Ramos Campagnoli, and Gabriela Guerra Leal Souza

Supplementary Material

Priming texts

Training block priming text: "The history of the human species evolution is marked by the gradual development of the ability to handle and make utensils and tools. Spears, knives, and gourds were probably the first objects manufactured by man. Assisting in daily household tasks or serving as decorative pieces, the objects have crossed the centuries. Even today they are part of everyday life in such an intense way that you can no longer live without them.”

Bonding block priming text: “Human beings are a social specie and, therefore, need to be together during both childhood and adulthood. In evolutionary terms, responding promptly to social cues guarantee the survival of the species, as it allows the maintenance of social bonds, which are very important for reproduction, food localization, and defense. In this way, individuals have a strong intrinsic motivation to form emotional bonds and share experiences.”

Control block priming text: “Currently, the lack of time, stress, and the excessive use of new technologies have caused a reduction in visual and social contact between individuals, contrary to the social nature of the human species. This reduction has been building since childhood, in the relationships between parents and children, and even among the children themselves, who in many moments, despite being close to each other, prefer to do activities individually.”

**Table S1**

*Skewness, kurtosis and Shapiro-Wilk test of normality for electromyographic activity of zygomaticus major and corrugator supercilli muscles, trait and state scales data from study 1 and study 2.*

|  | **Variable** | **Study 1** | | | | | | **Study 2** | | | | | |
| --- | --- | --- | --- | --- | --- | --- | --- | --- | --- | --- | --- | --- | --- |
|  |  | **Skewness** | **Skewness Standard Error** | **Kurtosis** | **Kurtosis Standard Error** | **Shapiro-Wilk (W)** | **p-value** | **Skewness** | **Skewness Standard Error** | **Kurtosis** | **Kurtosis Standard Error** | **Shapiro-Wilk (W)** | **p-value** |
| **Zygomaticus major** | Median EMG activity bonding pictures | 2.44 | 0.28 | 7.06 | 0.56 | 0.70 | <0.00001* | 1.08 | 0.28 | 0.66 | 0.55 | 0.88 | 0.0001* |
|  | Median EMG activity control pictures | 1.85 | 0.28 | 5.76 | 0.55 | 0.81 | <0.00001* | -1.41 | 0.28 | 4.40 | 0.55 | 0.88 | <0.00001* |
| **Corrugator suppercilli** | Median EMG activity bonding pictures | -0.92 | 0.27 | 2.87 | 0.54 | 0.89 | <0.00001* | -0.79 | 0.28 | 0.77 | 0.56 | 0.94 | 0.002* |
|  | Median EMG activity control pictures | -0.89 | 0.27 | 2.89 | 0.54 | 0.91 | 0.00004* | -0.78 | 0.28 | 2.72 | 0.54 | 0.90 | 0.0002* |
| **Trait Scales** | Mutual Grooming | 0.82 | 0.28 | 0.54 | 0.55 | 0.95 | 0.005* | 0.54 | 0.27 | -0.32 | 0.54 | 0.96 | 0.01* |
|  | IRI - Global Empathy | -0.77 | 0.28 | 0.85 | 0.55 | 0.96 | 0.012* | -0.27 | 0.27 | -0.18 | 0.54 | 0.98 | 0.31 |
|  | Emotional Contagion | -0.23 | 0.28 | 0.02 | 0.55 | 0.99 | 0.84 | - | - | - | - | - | - |
| **Mood State Scales** | Hope for closeness (moment A) | -0.56 | 0.27 | 0.61 | 0.53 | 0.97 | 0.13 | -0.08 | 0.27 | -0.29 | 0.53 | 0.99 | 0.51 |
|  | Hope for closeness (moment B) | -0.60 | 0.27 | 0.63 | 0.53 | 0.97 | 0.10 | -0.46 | 0.27 | -0.21 | 0.53 | 0.97 | 0.06 |
|  | Hope for closeness (moment C) | -0.48 | 0.27 | 0.39 | 0.53 | 0.98 | 0.20 | -0.13 | 0.27 | -0.24 | 0.53 | 0.97 | 0.11 |
|  | Fear of rejection (moment A) | 1.40 | 0.27 | 3.28 | 0.53 | 0.90 | 0.00002* | 0.46 | 0.27 | -0.39 | 0.53 | 0.97 | 0.03* |
|  | Fear of rejection (moment B) | 1.03 | 0.27 | 2.26 | 0.53 | 0.94 | 0.002* | 0.45 | 0.27 | -0.23 | 0.53 | 0.97 | 0.07* |
|  | Fear of rejection (moment C) | 0.90 | 0.27 | 1.15 | 0.53 | 0.95 | 0.004* | 0.39 | 0.27 | -0.94 | 0.53 | 0.94 | 0.001* |
|  | Altruistic behavior (moment A) | -0.67 | 0.27 | 0.75 | 0.53 | 0.96 | 0.02* | -0.67 | 0.27 | 0.29 | 0.53 | 0.96 | 0.008* |
|  | Altruistic behavior (moment B) | -0.95 | 0.27 | 1.74 | 0.53 | 0.95 | 0.003* | -0.52 | 0.27 | -0.13 | 0.53 | 0.97 | 0.045* |
|  | Altruistic behavior (moment C) | -0.37 | 0.27 | -0.21 | 0.53 | 0.98 | 0.30 | -0.85 | 0.27 | 0.82 | 0.53 | 0.95 | 0.005* |

*stands for non-normally distributed data. IRI: Interpersonal Reactivity Index

**Table S2**

*Post-hoc(within block) comparisons between time bins for the zygomatic major and corrugator supercilii electromyographic activities for bonding and control pictures in Study 1 and Study 2.*

| **Study 1** |  | **Zygomaticus Major** | | **Corrugator Supercilii** | | | |
| --- | --- | --- | --- | --- | --- | --- | --- |
|  |  | **Bonding Pictures** | | **Bonding Pictures** | | **Control Pictures** | |
|  | Interval | Z | p-value | Z | p-value | Z | p-value |
|  | 0.5 s - 1.0 s | 2.84 | 0.004* | 4.51 | <0.0001* | 3.38 | 0.0007* |
|  | 1.0 s - 1.5 s | 2.86 | 0.004* | 0.33 | 0.74 | 0.32 | 0.75 |
|  | 1.5 s - 2.0 s | 2.32 | 0.02* | 2.91 | 0.004* | 1.10 | 0.27 |
|  | 2.0 s - 2.5 s | 0.89 | 0.37 | 0.53 | 0.60 | 0.85 | 0.40 |
|  | 2.5 s - 3.0 s | 0.27 | 0.79 | 2.67 | 0.007* | 0.92 | 0.36 |
|  | 3.0 s - 3.5 s | 1.61 | 0.11 | 0.50 | 0.62 | 0.97 | 0.33 |
|  | 3.5 s - 4.0 s | 2.06 | 0.039* | 0.87 | 0.39 | 0.43 | 0.67 |
|  | 4.0 s - 4.5 s | 1.92 | 0.05 | 1.35 | 0.18 | 0.87 | 0.39 |
|  | 4.5 s - 5.0 s | 0.95 | 0.34 | 1.55 | 0.12 | 0.08 | 0.93 |
|  | 5.0 s - 5.5 s | 0.81 | 0.42 | 0.24 | 0.81 | 2.04 | 0.04* |
|  | 5.5 s - 6.0 s | 2.77 | 0.006* | 0.49 | 0.63 | 0.69 | 0.49 |
|  | 6.0 s - 6.5 s | 2.87 | 0.004* | 1.97 | 0.048* | 1.86 | 0.06 |
|  | 6.5 s - 7.0 s | 0.84 | 0.40 | 0.12 | 0.91 | 0.01 | 0.99 |
|  | 7.0 s - 7.5 s | 0.70 | 0.49 | 1.01 | 0.31 | 1.15 | 0.25 |
|  | 7.5 s - 8.0 s | 1.16 | 0.25 | 1.52 | 0.13 | 0.36 | 0.72 |
| **Study 2** |  | **Zygomaticus Major** | | | | **Corrugator Supercilii** | |
|  |  | **Bonding Pictures** | | **Control Pictures** | | **Bonding Pictures** | |
|  | Interval | Z | p-value | Z | p-value | Z | p-value |
|  | 0.5 s - 1.0 s | 4.11 | 0.00004* | 1.53 | 0.13 | 5.23 | < 0.00001* |
|  | 1.0 s - 1.5 s | 4.74 | < 0.00001* | 2.17 | 0.03* | 2.18 | 0.03* |
|  | 1.5 s - 2.0 s | 3.26 | 0.001* | 1.36 | 0.17 | 1.11 | 0.27 |
|  | 2.0 s - 2.5 s | 0.12 | 0.90 | 0.82 | 0.41 | 0.59 | 0.56 |
|  | 2.5 s - 3.0 s | 3.08 | 0.002* | 2.40 | 0.016* | 1.89 | 0.06 |
|  | 3.0 s - 3.5 s | 1.95 | 0.051 | 3.21 | 0.001* | 0.20 | 0.85 |
|  | 3.5 s - 4.0 s | 2.07 | 0.039* | 0.81 | 0.42 | 1.81 | 0.07 |
|  | 4.0 s - 4.5 s | 2.38 | 0.017* | 1.60 | 0.11 | 0.97 | 0.33 |
|  | 4.5 s - 5.0 s | 0.99 | 0.32 | 0.42 | 0.67 | 0.26 | 0.79 |
|  | 5.0 s - 5.5 s | 1.52 | 0.13 | 0.28 | 0.78 | 1.22 | 0.22 |
|  | 5.5 s - 6.0 s | 1.80 | 0.07 | 1.13 | 0.26 | 1.15 | 0.25 |
|  | 6.0 s - 6.5 s | 4.78 | < 0.00001* | 2.23 | 0.026* | 1.86 | 0.06 |
|  | 6.5 s - 7.0 s | 3.04 | 0.002* | 2.26 | 0.024* | 0.38 | 0.70 |
|  | 7.0 s - 7.5 s | 1.03 | 0.30 | 0.37 | 0.71 | 0.02 | 0.99 |
|  | 7.5 s - 8.0 s | 2.46 | 0.014* | 0.32 | 0.75 | 2.53 | 0.01* |
|  | 8.0 s - 8.5 s | 0.72 | 0.47 | 0.01 | 0.99 | 1.60 | 0.11 |
|  | 8.5 s - 9.0 s | 0.35 | 0.73 | 0.49 | 0.62 | 0.80 | 0.42 |
|  | 9.0 s - 9.5 s | 2.61 | 0.009* | 0.35 | 0.73 | 1.80 | 0.07 |
|  | 9.5 s - 10.0 s | 2.55 | 0.011* | 1.44 | 0.15 | 1.90 | 0.06 |

*Note.* Post-hoc comparisons presented are between time bins from significant Friedman Analysis of Variance findings.

Study 1: Post-hoc analyzes for the EMG activity of zygomaticus major muscle are presented from comparisons during bonding block (χ² = 80.01; p < 0.0001). Zygomatic activity during the control block did not change (χ² = 10.99; p = 0.75). Post-hoc analyzes for the EMG activity of corrugator supercilii muscle are presented from comparisons during bonding block (χ² = 108.71; p < 0.0001) and control block (χ² = 37.19; p = 0.01).

Study 2: Post-hoc analyzes for the EMG activity of zygomaticus major muscle are presented from comparisons during bonding (χ² = 188.44; p < 0.00001) and control blocks (χ² = 73.09; p < 0.00001). Post-hoc analyzes for the EMG activity of corrugator supercilii muscle are presented from comparisons during bonding block (χ² = 102.70; p < 0.00001). No differences were found during control block (χ² = 25.17; p = 0.15).

* stands for significant differences (p < 0.05).

**Table S3**

*Post-hoc comparisons between conditions (bonding and control pictures) for the zygomatic major and corrugator supercilii electromyographic activities in Study 1 and Study 2.*

| **Study 1** |  | **Zygomaticus Major** | | **Corrugator Supercilli** | |
| --- | --- | --- | --- | --- | --- |
|  | Time | Z | p-value | Z | p-value |
|  | 0.5 s | 0.89 | 0.37 | 0.21 | 0.83 |
|  | 1.0 s | 1.33 | 0.18 | 1.37 | 0.17 |
|  | 1.5 s | 1.78 | 0.08 | 0.72 | 0.47 |
|  | 2.0 s | 1.80 | 0.07 | 0.01 | 0.99 |
|  | 2.5 s | 2.52 | 0.012* | 0.32 | 0.75 |
|  | 3.0 s | 1.78 | 0.07 | 2.26 | 0.02* |
|  | 3.5 s | 3.48 | 0.0005* | 1.31 | 0.19 |
|  | 4.0 s | 2.58 | 0.01* | 2.79 | 0.005* |
|  | 4.5 s | 1.86 | 0.06 | 3.00 | 0.003* |
|  | 5.0 s | 1.98 | 0.047* | 2.58 | 0.010* |
|  | 5.5 s | 1.54 | 0.12 | 0.80 | 0.42 |
|  | 6.0 s | 1.12 | 0.26 | 1.01 | 0.31 |
|  | 6.5 s | 0.60 | 0.55 | 1.57 | 0.12 |
|  | 7.0 s | 1.82 | 0.07 | 2.01 | 0.044* |
|  | 7.5 s | 1.72 | 0.08 | 1.23 | 0.22 |
|  | 8.0 s | 0.73 | 0.46 | 0.51 | 0.61 |
| **Study 2** |  | **Zygomaticus Major** | | **Corrugator Supercilli** | |
|  | Time | Z | p-value | Z | p-value |
|  | 0.5 s | 1.01 | 0.31 | 0.07 | 0.94 |
|  | 1.0 s | 2.47 | 0.014* | 2.11 | 0.035* |
|  | 1.5 s | 3.99 | < 0.0001* | 1.97 | 0.048* |
|  | 2.0 s | 4.04 | < 0.0001* | 2.66 | 0.008* |
|  | 2.5 s | 4.70 | < 0.0001* | 2.78 | 0.005* |
|  | 3.0 s | 4.95 | < 0.0001* | 1.57 | 0.12 |
|  | 3.5 s | 4.98 | < 0.0001* | 1.99 | 0.046* |
|  | 4.0 s | 4.32 | < 0.0001* | 1.08 | 0.28 |
|  | 4.5 s | 3.78 | 0.0002* | 1.70 | 0.09 |
|  | 5.0 s | 3.64 | 0.0003* | 1.74 | 0.08 |
|  | 5.5 s | 3.78 | 0.0002* | 2.47 | 0.01* |
|  | 6.0 s | 3.98 | < 0.0001* | 1.66 | 0.10 |
|  | 6.5 s | 3.46 | 0.0005* | 2.80 | 0.005* |
|  | 7.0 s | 3.71 | 0.0002* | 1.17 | 0.24 |
|  | 7.5 s | 3.49 | 0.0005* | 2.26 | 0.02* |
|  | 8.0 s | 2.38 | 0.017* | 1.70 | 0.09 |
|  | 8.5 s | 2.38 | 0.017* | 1.53 | 0.13 |
|  | 9.0 s | 3.26 | 0.001* | 2.19 | 0.03* |
|  | 9.5 s | 2.98 | 0.003* | 3.10 | 0.002* |
|  | 10.0 s | 1.33 | 0.18 | 0.36 | 0.72 |

*Note.* Study 1: Post-hoc analyzes are presented for cross-time comparisons between bonding and control blocks for the zygomatic electromyographic activity (χ² = 108.61; p < 0.0001) and corrugator electromyographic activity (χ² = 122.36; p < 0.0001).

Study 2: Post-hoc analyzes are presented for cross-time comparisons between bonding and control blocks for the zygomatic electromyographic activity (χ² = 291.09; p < 0.00001) and corrugator electromyographic activity (χ² = 192.76; p < 0.00001).

* stands for significative differences (p < 0.05).

**Figure S1**

*Temporal course of the electromyographic activity (EMG) of (a) the zygomaticus major muscle and, (b) the corrugator supercilli muscles in Study 1.*

*
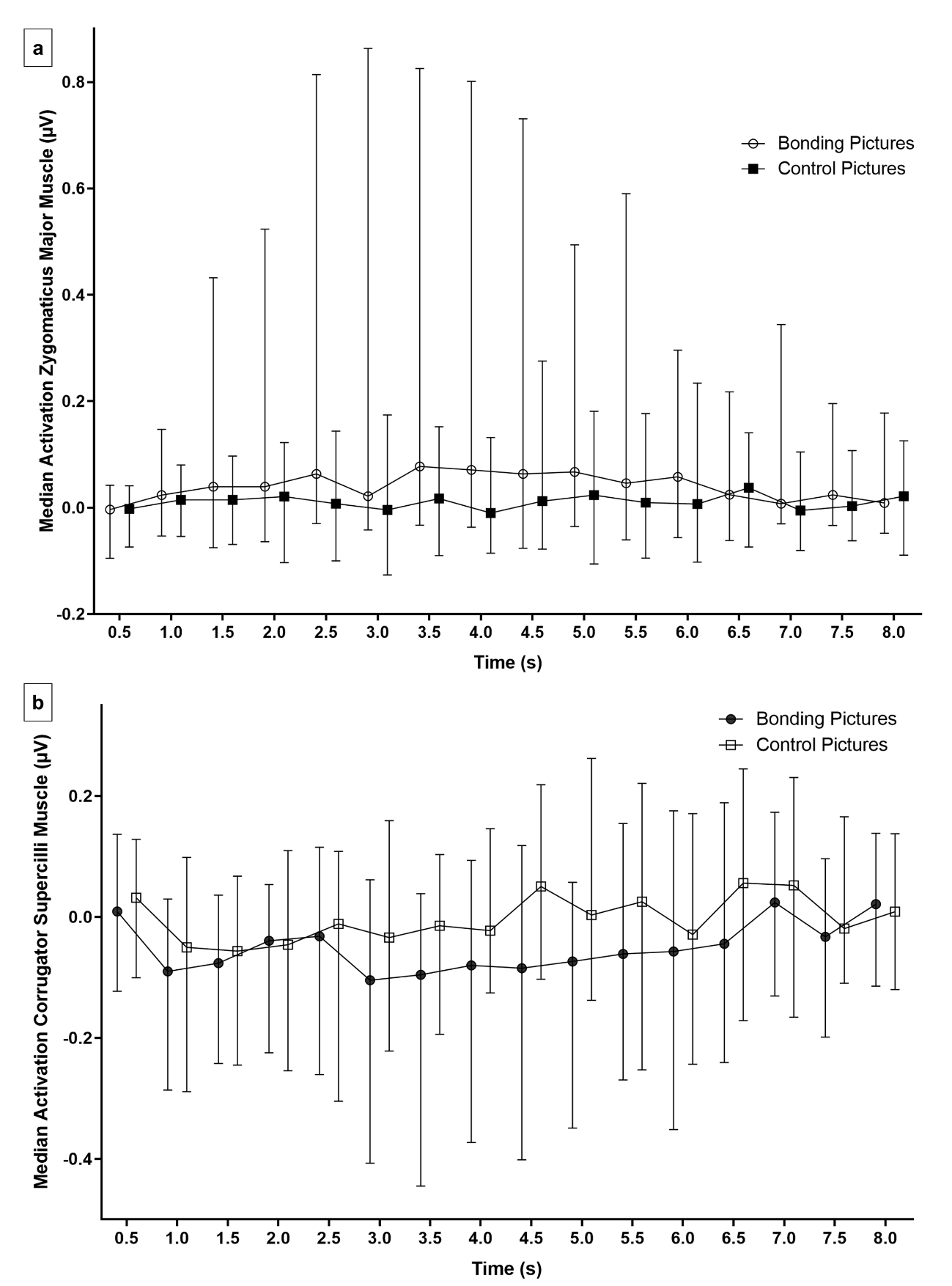
*

*Note.* The pictures were displayed on the screen between 0 and 4 seconds. From 4 seconds on, the intertrial interval occurs. Graphs display the entire analysis window. Error bars stand for the interquartile range, representing minimum and maximum values.

**Figure S2**

*Temporal course of the electromyographic activity (EMG) of (a) the zygomaticus major muscle and, (b) the corrugator supercilli muscle in Study 2.*

*
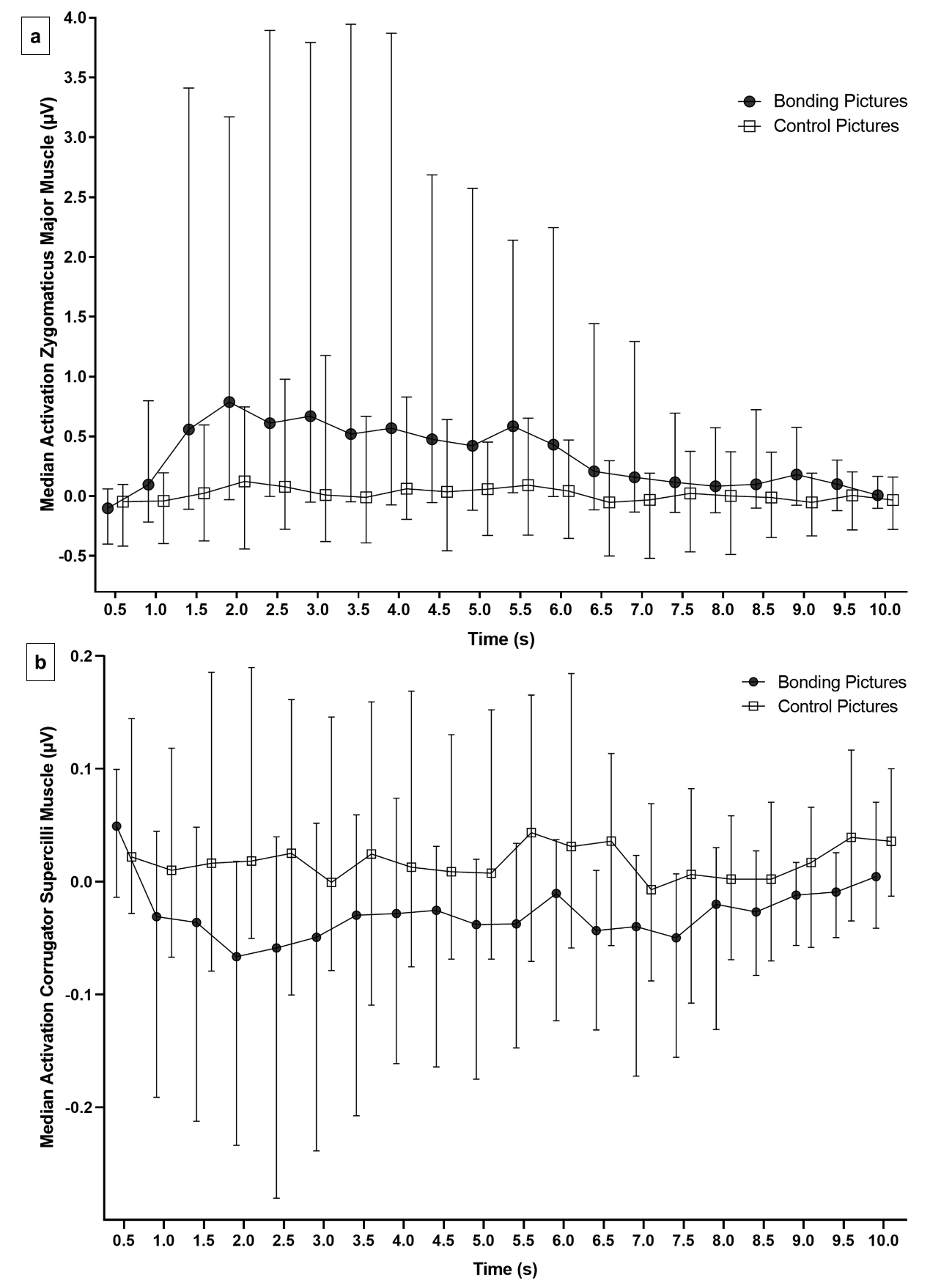
*

*Note.* The pictures were displayed on the screen between 0 and 6 seconds. From 6 seconds on, the intertrial interval occurs. Graphs display the entire analysis window. Error bars stand for the interquartile range, representing minimum and maximum values.

**Table S4**

*Correlation analyses between the EMG activities of the Zygomaticus Major and Corrugator Supercilii muscles during visualization of the Bonding and Control pictures and the scales of individual traits in Study 1 and Study 2.*

|  |  | **Zygomatic Amplitude** | | **Corrugator Amplitude** | |
| --- | --- | --- | --- | --- | --- |
| **Study 1** | **Bonding Pictures** | **Rho** | **p-value** | **Rho** | **p-value** |
|  | Emotional Contagion | 0.18 | 0.13 | -0.24 | 0.032 |
|  | IRI – Global Empathy | 0.29 | 0.012* | -0.19 | 0.089 |
|  | Mutual Grooming | 0.31 | 0.008* | -0.21 | 0.066 |
|  | **Control Pictures** | **Rho** | **p-value** | **Rho** | **p-value** |
|  | Emotional Contagion | 0.05 | 0.665 | -0.03 | 0.786 |
|  | IRI – Global Empathy | 0.17 | 0.152 | 0.004 | 0.969 |
|  | Mutual Grooming | 0.09 | 0.457 | 0.24 | 0.031 |
| **Study 2** | **Bonding Pictures** | **Rho** | **p-value** | **Rho** | **p-value** |
|  | IRI – Global Empathy | 0.28 | 0.01* | 0.004 | 0.970 |
|  | Mutual Grooming | 0.15 | 0.17 | 0.23 | 0.047 |
|  | **Control Pictures** | **Rho** | **p-value** | **Rho** | **p-value** |
|  | IRI – Global Empathy | 0.08 | 0.48 | -0.14 | 0.256 |
|  | Mutual Grooming | -0.014 | 0.90 | 0.08 | 0.531 |

*Note.* * stands for significant correlation after Bonferroni correction (Study 1: p < 0.017; Study 2: p < 0.025); IRI: Interpersonal Reactivity Index
